# One of the Triple Poly(A) Signals in the M112-113 Gene Is Important and Sufficient for Stabilizing the M112-113 mRNA and the Replication of Murine Cytomegalovirus

**DOI:** 10.3390/v12090954

**Published:** 2020-08-28

**Authors:** Ruth Cruz-cosme, Najealicka Armstrong, Qiyi Tang

**Affiliations:** Department of Microbiology, College of Medicine, Howard University, 520 W Street NW, Washington, DC 20059, USA; ruth.cruzcosme@Howard.edu (R.C.-c.); narmstrong@Howard.edu (N.A.)

**Keywords:** cytomegalovirus (CMV), M112-113, poly(A) signal (PAS), major immediate early (MIE), IE3, bacterial artificial chromosome (BAC), gene activation

## Abstract

The M112-113 gene is the first early gene of the murine cytomegalovirus (MCMV), and its expression is activated by the immediate-early 3 (IE3) protein during MCMV infection in permissive cells. At its 5′ terminus, a 10-bp motif, upstream of the TATA box of the M112-113 gene, was identified to bind to IE3, and it is necessary for IE3 to activate M112-113 gene expression (Perez KJ et al. 2013 JVI). At the 3′ terminus of the M112-113 gene, three poly(A) signals (PASs) are arranged closely, forming a PAS cluster. We asked whether it is necessary to have the PAS cluster for the M112-113 gene and wondered which PAS is required or important for M112-113 gene expression. In this study, we mutated one, two, or all three PASs in expressing plasmids. Then, we applied bacterial artificial chromosome (BAC) techniques to mutate PASs in viruses. Gene expression and viral replication were analyzed. We found that not all three PASs are needed for M112-113 gene expression. Moreover, we revealed that just one of the three poly(A)s is enough for MCMV replication. However, the deletion of all three PASs did not kill MCMV, although it significantly attenuated viral replication. Finally, an mRNA stability assay was performed and demonstrated that PASs are important to stabilize M112-113 mRNA. Therefore, we conclude that just one of the PASs of the M112-113 gene is sufficient and important for MCMV replication through the stabilization of M112-113 mRNA.

## 1. Introduction

Murine cytomegalovirus (MCMV) infection in mice is the most often used small animal model for investigating human CMV (HCMV) pathogenesis due to the similarities between HCMV and MCMV [1]. CMV belongs to the beta-subfamily of the *Herpesviridae* family, and it contains an about-235-kb DNA genome that encodes about 200 genes [2]. CMV gene expression is sequentially regulated in the infected cells, and CMV genes are accordingly defined as immediate-early (IE), early (E), and late (L) genes [2]. IE gene expression is independent of any viral de novo gene expression; E gene expression depends on IE proteins, and early proteins are important for viral DNA replication and late gene expression [2]. The mechanism of how IE proteins regulate E gene expression has been a focus of studies in the CMV field, and it is still elusive.

The M112-113 gene is thought to be the first gene expressed at the early stage (i.e., after the immediate early stage) after MCMV infection in permissive cells, so it is also called E1 (early protein 1) [3]. Its homolog in HCMV is UL112-113, and UL112-113 protein is one of the eleven core proteins that are essential for HCMV DNA replication [4,5]. The M112-113 gene has drawn a lot of attention recently because of its important biological functions. First, the M112-113 gene is silent during latency, and it might be the first activated gene when switching to lytic infection [1]. Therefore, M112-113 might be critical for the progression of viral reactivation to viral particle formation. Second, the M112-113 promoter was specifically activated in neuronal cells (not in non-neuronal cells) in a transgenic mouse strain, which implies that M112-113 might be important for CMV persistent infection in the brain [6]. Third, UL112-113 (the HCMV homolog) was found to be able to reactivate KSHV from latency [7], which suggests the clinical importance of CMV in mixed herpes viral infection. In addition, it was recently found that M112-113 proteins were important for MCMV to productively infect human cells [8], which also raises a clinical concern with regard to the possibility of MCMV infection in human populations. Furthermore, M112-113 proteins have evolved functions to inhibit IE3’s repressive effects on the major immediate early promoter (MIEP) so that MCMV can more efficiently express its genes and replicate [9]. In a transfection system using M112-113-expressing plasmid, where the M112-113 gene is under the control of its own promoter, we found that M112-113 proteins can be expressed at a low level without IE3, but IE3 enhanced the M112-113 gene expression to a much higher level [9].

The M112-113 gene consists of three exons, and the alternative splicing gives rise to at least 4 proteins (33, 36, 38, and 87 kDa) [3,10,11]. Earlier studies of HCMV UL112-113 gene regulation by IE2 (the equivalent of MCMV IE3) have indicated that a minimal DNA sequence containing an ATF/CREB-binding motif is important for IE2 to activate UL112-113 promoter activity [12]. Further, it was confirmed that IE2 mediated the transactivation of the UL112-113 promoter via interactions with CREB and CREB-binding protein (CBP) [13,14]. It was reported that three binding sites exist for IE2, upstream of the UL112-113 promoter, but those binding sites were found not to be critical for IE2 to activate the UL112-113 gene [13,15]. More interestingly, Rodems and coworkers discovered that cis-elements containing the ATF/CREB-binding motif and IE86-binding motif differentially activate the UL112-113 promoter at early and late times in HCMV infection [14]. M112-113 and UL112-113 have similar gene structures [11,16], but M112-113 lacks the ATF/CREB-binding motif.

Interestingly, there are three PASs arranged closely as a cluster in the 3′ terminus of the M112-113 gene, which is not found in the UL112-113 gene of HCMV. Therefore, the model of IE2 activation of UL112-113 in HCMV may not be simply applied to the mechanism of IE3 activation of M112-113. We are curious whether all the three PASs are important for M112-113 gene expression. We found that one of the triple poly(A) signals is enough for M112-113 gene expression, and the PASs are important for MCMV replication.

## 2. Materials and Methods

### 2.1. Tissue Culture

NIH3T3 cells (ATCC) were maintained in Dulbecco’s modified Eagle’s medium (DMEM) supplemented with 10% fetal calf serum (FCS), penicillin (100 IU/mL)–streptomycin (100 µg/mL), and amphotericin B (2.5 µg/mL) [17].

### 2.2. Antibodies

The antibodies used for Western blot (WB) are listed, and their dilutions are shown below. The monoclonal antibody against GAPDH (sc-365062, 1:500 for WB) and the monoclonal against GFP (sc-9966, 1:1000 for WB) were purchased from Santa Cruz Biotechnology (Santa Cruz, CA, USA); monoclonal antibodies against MCMV IE1 and M112-113 proteins were provided by Dr. Stipan Jonjic (Medicine University of Rijeka, Rijieka, Croatia; 1:200 for WB) [9,18]; the monoclonal antibody against M25 was a generous gift from Dr. Shanley (University of Connecticut., Stamford, CT, USA; 1:250 for WB) [19,20].

### 2.3. Immunofluorescent Assay (IFA)

Immunostaining was performed on cells grown on coverslips after fixation with 1% paraformaldehyde (10 min at room temperature) and permeabilization in 0.2% Triton (20 min on ice) by sequential incubation with primary and Texas red (TR)-labeled secondary antibodies (Vector Laboratories, Burlingame, CA, USA.) for 30 min each (all solutions in PBS). Finally, cells were equilibrated in PBS, stained for DNA with Hoechst 33258 (0.5 μg/mL), and mounted in Fluoromount G (Fisher Scientific, Newark, DE, USA).

### 2.4. Plasmids and Molecular Cloning

**Figure 1:**
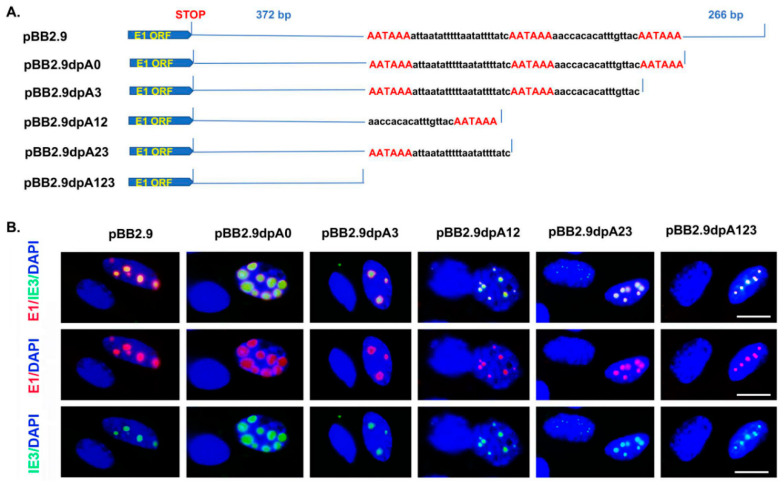
Construction of plasmids with mutation of the poly(A) signal cluster. (**A**) The diagram of the M112-113 gene and resultant mutations at the end of the M112-113 gene. E1 ORF stands for M112-113 coding open reading frame. Red words represent the poly(A) signal in the 3′ terminus of the M112-113 gene. (**B**). Immunofluorescent assay to show M112-113 and GFPIE3 proteins after cotransfection of M112-113 expressing plasmids with GFPIE3-expressing plasmid for 24 h in NIH3T3 cells. M112-113 protein is stained with Texas red (TR) and IE3 is shown in green. Scale bar: 10 μm.

The plasmid pBB2.9, containing the whole M112-113 gene and the promoter, has been previously described [21]; pET (expressing GFP) and pgfpie3 (expressing GFP-tagged IE3) have been previously reported [22]. A diagram of part of the M112-113 gene and the constructed M112-113-expressing vectors is shown in Figure 1A. pBB2.9 consists of a 300-bp DNA sequence before the first amino acid (aa) code (ATG), a 1.9-kb M112-113 coding sequence (including exons and introns), and a 700-bp UTR after the stop codon. Using this plasmid as the template, we performed a PCR and generated 5 DNA fragments with different mutations of the poly(A) cluster at the end of the DNA sequence (Figure 1A). Primers were designed to include ClaI and PstI restriction sites at the ends (Table 1). The amplified DNA fragments by overlapping PCR were cloned into the ClaI and PstI sites of pBB2.9, resulting in vectors pBB2.9dlpA0, pBB2.9dpA3, pBB2.9dlpA12, pBB2.9dlpA23, and pBB2.9dlpA123 (Figure 1A).

### 2.5. Bacterial Artificial Chromosome (BAC) Mutagenesis to Generate MCMVs with Mutated E1 Poly(A)

The MCMV BACmid DNA (Sm3frE5gfp) was made from BACmid Sm3fr [23] by tagging GFP to the C-terminus of IE3 (end of exon 5) [22]. Based on Sm3frE5gfp, we further mutated the DNA sequence in the poly(A) region of the M112-113 gene. The seamless BAC system, using galK as the selection marker [22,24], was utilized to construct the mutant and the revertant (Figure 4A); the primers for the mutagenesis are summarized in Table 2. First, the DNA sequence, including the M112-113 poly(A) cluster, was replaced with the galK gene by PCR using primers E1polyA_galK_Fw and E1polyA_galK_Rv (Table 2). Then, the galK gene was replaced by DNA fragments by PCR using the E1polyA_Fw primer and the reverse primer, as shown in Table 2. We set out to delete one, two, or all three poly(A) signals. The resultant BAC DNAs were named Sm3frE5gfp_dlpA3, Sm3frE5gfp_dlpA23, and Sm3frE5gfp_dlpA123. To generate the viruses from BAC DNAs, the BAC DNAs were transfected into NIH3T3 cells. Positive viral plaques were picked and propagated in NIH3T3 cells to make larger volumes of viral stocks. The resultant MCMVs were named MCMVE5gfp_dpA3, MCMVE5gfp_dpA23, and MCMVE5gfp_dpA123. To make revertant viruses, the poly(A) part was replaced by wild-type poly(A) sequences, and the viruses were named MCMVE5gfp_dpA3RV, MCMVE5gfp_dpA23RV, and MCMVE5gfp_dpA123RV.

### 2.6. RNA Isolation, Treatment with DNase I (RNase Free), and Real-Time RT-PCR

Total RNA from the NIH3T3 cells was isolated using TRI Reagent (Ambion, Inc., Austin, TX, USA) and treated with DNase I (RNase-free, Invitrogen, Cat# 18047-019) to remove any remaining DNA, according to the manufacturer’s instructions. DNase I was then inactivated by treating the RNA sample with phenol and chloroform isolation. RT-PCR and/or real-time PCR was performed using primers to amplify the M112-113 gene.

To quantitatively examine the mRNA level of M112-113 in M112-113-transfected or MCMV-infected cells, a real-time RT-PCR was performed using the SsoAdvanced Universal SYBR Green Supermix kit (Bio-Rad, Hercules, CA, USA). A total of 1 ug of total RNA and 0.2 uM of sense and antisense primers (Table 3) were used in a final 25 uL master mix volume. A reverse transcription step of 20 min at 50 °C was included using the iScript cDNA kit (Bio-Rad, Hercules, CA, USA) prior to PCR. PCR reactions consisted of 50 cycles with optimal conditions as follows: 94 °C for 20 s, 50 °C for 1 min, 72 °C for 30 s, and an optimized collection data step, 80 °C for 5 s. Fluorescence captured at 80 °C was determined to be absent of signals generated by primer dimers. All samples were run in triplicate; data were collected and recorded by CFX Manager software (Bio-Rad) and expressed as a relative fold expression of the cycle threshold (CT), which represents the number of cycles at which the fluorescent intensity of the SYBR Green dye is significantly greater than the background fluorescence. A melting temperature curve analysis was obtained by measuring the fluorescence during a period of warming from 60 to 95 °C after the amplification cycles. Data were collected, and the relative gene expression level was analyzed by the 2^−ΔΔCT^ method using CFX software (Bio-Rad) using GADPH as the reference gene [17].

### 2.7. Immunoblot Analysis

To determine the levels of MCMV proteins, the whole-cell lysates were separated by SDS-PAGE in 7.5% polyacrylamide gels (10 to 20 µg loaded in each lane). The separated proteins in the gel were transferred to nitrocellulose membranes (Amersham Inc., Piscataway, NJ, USA) and blocked with 5% nonfat milk for 60 min at room temperature. Membranes were incubated with a primary antibody, followed by an incubation with a horseradish peroxidase-coupled secondary antibody. For regular WB, we used a secondary antibody from Amersham. Detection was accomplished with enhanced chemiluminescence (Pierce, Rockford, IL, USA) according to standard methods. Membranes were stripped with stripping buffer (100 mM β-mercaptoethanol, 2% SDS, 62.5 mM Tris-HCl, pH 6.8), washed with PBS-0.1% Tween 20, and reprobed with new primary antibodies to detect additional proteins.

### 2.8. Plaque Formation Unit (PFU) Assay

The plaque formation unit (PFU) assay was used to determine viral titers, largely as described [22], but with a slight modification. Supernatants containing serially diluted virus particles were added to confluent NIH3T3 cell monolayers in 6-well plates. After adsorption for 2 h, the medium was removed, and the cells were washed twice with serum-free DMEM and overlaid with phenol-free DMEM containing 5% FCS, 0.5% low-melting-point agarose (GIBCO), and 1% penicillin–streptomycin. Then, plaques were stained with neutral red to enhance plaque visualization, and the plaque numbers were counted and calculated in 1 mL, which is pfu per ml. The mean pfu was determined after averaging from different dilutions.

### 2.9. M112-113 mRNA Stability Assay

To know whether the poly(A) signals are important for the stability of M112-113 mRNA, we performed an mRNA stability assay according to previously reported protocol [25]. Briefly, we infected NIH3T3 cells with MCMVE5gfp_dpA3, MCMVE5gfp_dpA23, or MCMVE5gfp_dpA123 and their revertant at an MOI (multipilicity of infection) of 0.1. At the time of 20 h postinfection (hpi), 10 μg/mL actinomycin D was added to cells. The cells were collected for isolation of total RNA at time points of 1, 2, 4, and 8 h following the actinomycin D addition. RT-qPCR was performed, as described above, for the M112-113 gene and GAPDH (Table 3). The levels of M112-113 mRNA were normalized with GAPDH. Then, the normalized M112-113 mRNA levels of MCMVE5gfp_dpA3, MCMVE5gfp_dpA23, or MCMVE5gfp_dpA123 were compared to the revertant of each mutated MCMV. The experiments were independently performed 3 times, and the averaged numbers were statistically compared using Student’s *t*-test.

## 3. Results

### 3.1. One of the POLY(A) SIGNALS Is Enough for IE3 to Activate M112-113 Gene Expression

Although the conserved sequences AAUAAA and AUUAAA cover a majority of PASs in mammalian genes [26], 10 other alternatives (AGUAAA, UAUAAA, CAUAAA, GAUAAA, AAUAUA, AAUACA, AAUAGA, ACUAAA, AAGAAA, AAUGAA) exist [27]. At the end of the M112-113 gene of MCMV, 372 bp downstream of the stop codon (TGA), there are 3 poly(A) signals (PASs) forming a PAS cluster: TGTAAATAAAATTAATATTTTTAA TATTTTATCAATAAAAACCACACATTTGTTACAATAAACACG. We wondered whether all three poly(A) signals (AATAAA) are needed for the expression of the M112-113 gene.

In doing so, we mutated the PAS cluster by deleting one, two, or all three PASs, resulting in the following plasmids: pBB2.9dlpA0, pBB2.9dlpA3, pBB2.9dlpA12, pBB2.9dlpA23, and pBB2.9dlpA123 (Figure 1A). To determine whether the constructed plasmids could positively express the M112-113 gene, we cotransfected the M112-113 plasmids (Figure 1A), together with the IE3-expressing plasmid, to NIH3T3 cells for 24 h. The cells were fixed to examine the proteins of M112-113 and GFP-tagged IE3 by immunofluorescent assay (IFA). As shown in Figure 1B, all the M112-113-expressing plasmids can translate the M112-113 gene, whose protein was immuno-stained with Texas red. GFP-tagged IE3 is shown in green, and it colocalized with M112-113. The colocalization of M112-113 with IE3 has been previously demonstrated by different laboratories [8,9,10,21,22]. Therefore, the M112-113 gene can be expressed even when we delete all three poly(A) signals.

**Figure 2:**
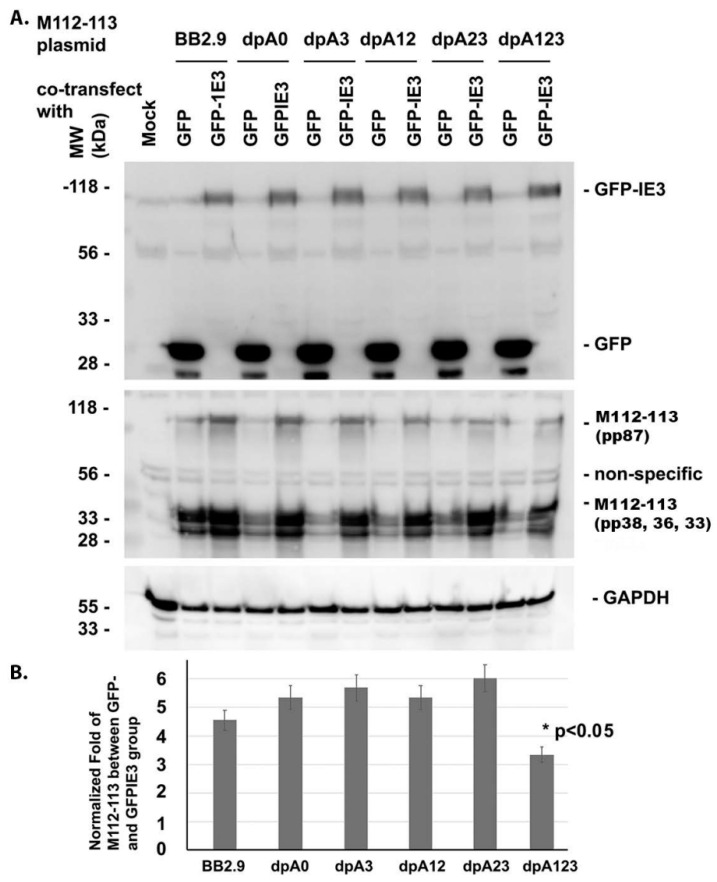
Western blot assay to determine the activation of M112-113 by immediate-early 3 (IE3). (**A**) The M112-113 plasmids were cotransfected with either GFP- or GFPIE3-expressing plasmid into NIH3T3 cells for 24 h. The whole-cell lysates were collected for a Western blot assay using antibodies against GFP, M112-113, or GAPDH to examine the protein as marked on the right side. One representative blot of three experiments is shown. (**B**) We have performed three independent experiments; the densitometry was obtained by averaging the three Western blots. The densities of the protein bands of M112-113 were first normalized with GAPDH and then compared between the GFPIE3 group and the GFP group. The ratios from 3 independent experiments are shown as a bar graph at the bottom. A Student’s *t*-test was applied to statistically analyze the data, and the significance was only seen for the group of dpA123 when compared to the other groups: * *p* < 0.05.

Since the activation of the M112-113 gene expression requires an immediate-early (IE) protein, IE3, we next asked whether the poly(A) signals were needed for the M112-113 gene to be activated by IE3. For that purpose, we cotransfected M112-113-expressing plasmids, together with either GFP-tagged IE3 [21] or GFP as a control, into NIH3T3 cells. The whole-cell lysate samples were collected after 24 h post-transfection and used for Western blot assays. An enhancement of M112-113 protein by GFP-IE3 rather than GFP suggests the activation of the M112-113 gene by IE3. As shown in Figure 2, the M112-113 gene in all the vectors was activated by IE3 because the M112-113 protein level in the GFP-tagged IE3 group was higher than that in the GFP group. The M112-113 gene encodes 4 proteins: pp87, pp38, pp36, and pp33, as marked at the right side of Figure 2. In our experiments, as shown in Figure 2, GAPDH was used as a sample-loading control, and a mock-infected cell group was used as a control for the viral proteins and GFP. The Western blot assay experiments were performed independently 3 times, and one of the results was selected for Figure 2A. To semiquantify the activation of the M-112-113 gene by IE3, we first normalized the M112-113 bands to their respective GAPDH. The normalized ratio was then compared between the GFPIE3 group and the GFP group. The average results of the 3 experiments are shown in Figure 2B. We compared the normalized fold expression of the M112-113 protein between the group of pBB2.9 and the other groups. Not surprisingly, the deletion of all the three poly(A) signals (dpA123) caused significantly less activated M112-113 gene expression by IE3 (shown as * *p* < 0.05). In summary, our experimental results in the context of the transfection system demonstrate that the poly(A) cluster is not required for M112-113 gene expression and is not needed for the M112-113 gene to be activated by IE3.

**Figure 3:**
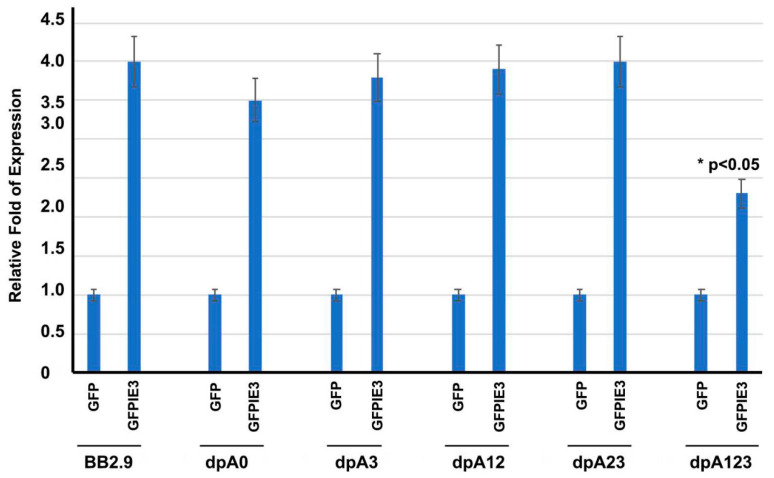
RT-qPCR assay to determine the activation of M112-113 by IE3. The M112-113 plasmids were cotransfected with either GFP- or GFPIE3-expressing plasmid into NIH3T3 cells for 24 h. The total RNA was isolated for an RT-qPCR assay using primers for M112-113 or GAPDH to examine mRNA levels. The M112-113 mRNA levels were first normalized with GAPDH and then compared between the GFPIE3 group and the GFP group. The ratios from 3 independent experiments are shown as a bar graph. A Student’s *t*-test was applied to statistically analyze the data, and significance was only seen for the group of dpA123 when compared to other groups: * *p* < 0.05.

Gene transcription starts from DNA in the nucleus to make mRNA, and the mRNA is exported to the cytoplasm for translation of a protein. The poly(A) signal is believed to be important for the exportation and stabilization of mRNA. We wondered whether the M112-113 mRNA could be affected by deleting the poly(A) signals. To that end, we cotransfected the M112-113 vectors with either the GFP-tagged IE3 plasmid or the GFP plasmid into NIH3T3 cells for 24 h. Then, the total RNA was isolated for performing an RT-qPCR to determine the mRNA level of the M112-113 gene. As shown in Figure 3, IE3 can activate the M112-113 gene transcription from all the plasmids. The M112-113-mRNA levels induced by IE3 is the least in the plasmid in which all the three poly(A) signals were deleted, which is consistent with the Western blot results (Figure 2). It is also consistent with Figure 2, where the deletion of the three PASs resulted in a significant reduction of mRNA of M112-113, as shown by the * in Figure 3.

### 3.2. Deletion of the M112-113 Poly(A) Signal Cluster Lessens MCMV Replication

**Figure 4:**
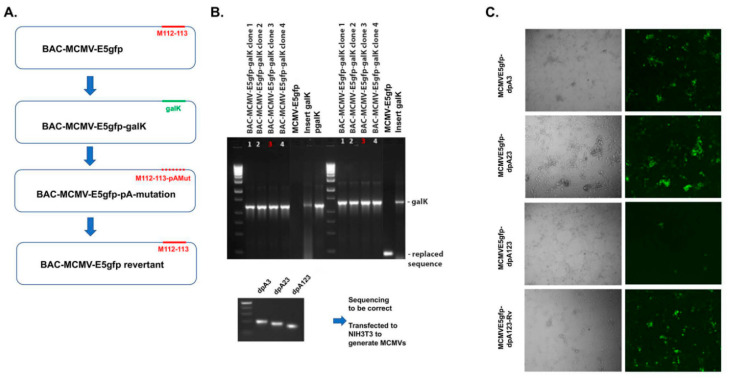
BAC mutagenesis to make mutant murine cytomegalovirus (MCMV). (**A**) A scheme of mutagenesis by BAC of MCMV. (**B**) PCR and DNA sequencing to identify the resultant BAC DNA. (**C**) BAC DNAs were transfected into NIH3T3 to generate MCMV mutants. The green dot-like cells show the formed plaques because the IE3 was tagged with GFP at its C-terminus of exon 5. The image was taken under a 10× magnificstion len of the microscope.

Although we have demonstrated that M112-113 gene expression is independent of the poly(A) signal cluster, the results were from the transfection system. We needed to determine whether it was needed for MCMV replication. For that purpose, we used bacterial artificial chromosome (BAC) technology [24] to mutate the poly(A) cluster in the MCMV genome. As shown in Figure 4A that depicts the workflow of the BAC mutagenesis based on Sm3frMCVME5gfp [21], the M112-113 gene was first replaced by a negative-to-positive selection marker, the galK gene. Then, the galK gene was replaced by the mutated M112-113 gene that had either deletion of one poly(A) signal (dpA3), two poly(A) signals (dpA23), or three poly(A) signals (dpA123). The generated mutant BAC DNAs were verified by PCR and sequencing to be correct (Figure 4B). Finally, the BAC DNAs were transfected into NIH3T3 cells to generate MCMV mutants, as shown in Figure 4C: MCMVE5gfp-dpA3, MCMVE5gfp-dpA23, and MCMVE5gfp-dpA123, respectively. In addition, we also made revertant viruses from each mutant by replacing the mutated M112-113 gene with the wild-type M112-113 gene: MCMVE5gfp-dpA3RV, MCMVE5gfp-dpA23RV, and MCMVE5gfp-dpA123RV.

Since IE3 was tagged with GFP at the end of exon 5, the cells with the replicated MCMV were visualized under an immunofluorescent microscope. As can be seen in Figure 4C, viral plaques formed in the NIH3T3 cells after the transfection. Interestingly, MCMVE5gfp-dpA123-transfected cells formed less and smaller plaques, while revertant BAC DNA that was transfected produced more and larger plaques. These were only from the first round of transfection of BAC DNAs when the attempts were only to generate viral stock. Therefore, we made more viral propagations for further accurate assays to determine whether the poly(A) is needed for MCMV replication and gene expression.

### 3.3. One M112-113 Poly(A) Signal Is Sufficient for MCMV to Productively Replicate

**Figure 5:**
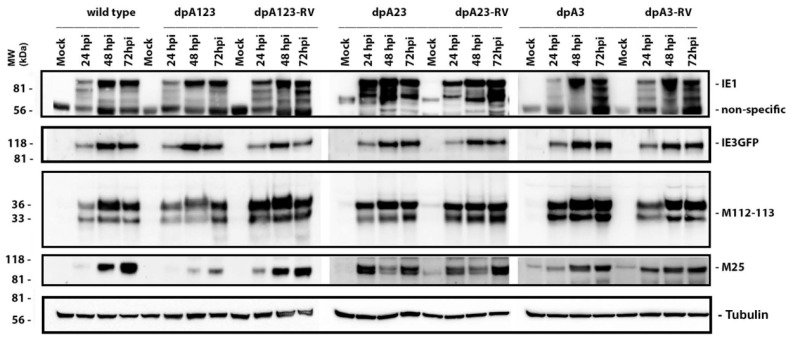
Viral protein production. NIH3T3 cells were infected with MCMVs (mutation, revertant, and wild-type) at an MOI of 0.1, and the whole-cell lysates were collected at the indicated time postinfection. The samples were loaded to run on SDS-PAGE, and the proteins were transferred to a nitrocellulose membrane for Western blot assay using the antibodies, as shown on the right side. Three independent experiments were performed, and one of the experiments is shown.

To determine whether any poly(A) signals are needed for viral protein production, we infected NIH3T3 cells with the wild-type (wt) poly(A) deletion (dpA) and its revertant virus (RV) at an MOI of 0.1. The cell lysate samples were collected at the indicated time, as shown on the top of Figure 5. Mock infection was used as a control. As can be seen, only the MCMVE5gfp-dpA123 infection showed decreased yields of M112-113 (very early protein) and M25 (late early protein), but the decrease in the expression of these proteins was not significantly seen in the infection group of MCMVE5gfp-dpA23 or -dpA3. As expected, IE1 and IE3 production were not significantly affected. More than one band of IE1 is probably caused by the alternative splicing of the MIE gene [21]. These Western blot results demonstrate that the poly(A) signals are needed for MCMV to have a similar amount of viral protein production as observed in wt MCMV.

**Figure 6:**
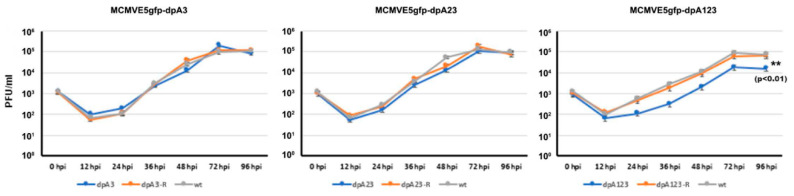
Viral growth curve by plaque formation unit (PFU) assay. The MCMVs (mutation, revertant, and wild-type) were infected onto NIH3T3 cells at an MOI of 0.01. The whole-cells and medium were collected at the indicated time postinfection. The samples were frozen and thawed for 3 cycles, and the supernatants were used for the PFU assay. Three independent experiments were performed, and the averaged PFU/mL numbers were used to create a viral growth curve. A *t*-test was used to compare the differences between the groups, ** *p* < 0.01.

To know whether the deletion of poly(A) signal(s) has any effect on viral replication, we infected NIH3T3 cells with the mutant viruses at an MOI of 0.01. Then, the viral samples (cells and medium) were collected at the indicated time, as shown in Figure 6. After releasing the viral particles from the cells, the supernatants were used for the PFU assay. The experiments were performed three times, and the average numbers were applied to generate the viral growth curve, as shown in Figure 6. Although we did not see any significant differences between the wild-type MCMV and the one or two PAS deletion MCMVs (MCMVdpA23 or MCMVdpA3), the deletion of all three PASs (MCMVdpA123) did cause significant attenuation of viral growth. Therefore, we discovered that the PASs are not essential for MCMV replication, and one PAS is needed for MCMV to have normal replication.

### 3.4. One M112-113 Poly(A) Signal Is Needed and Sufficient to Stabilize the M112-113 mRNA

**Figure 7:**
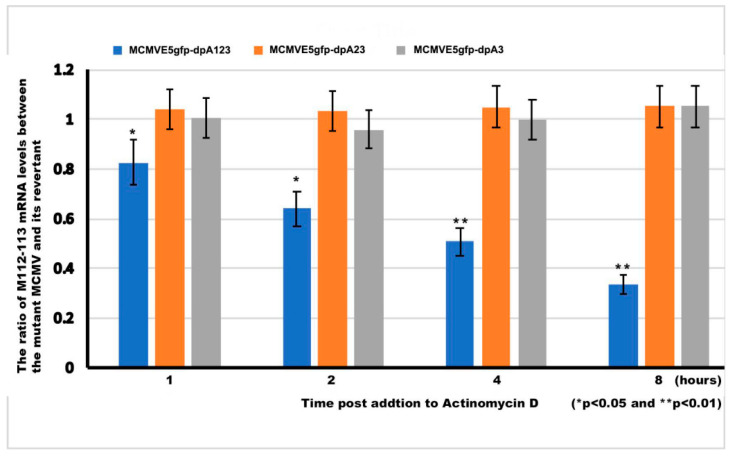
M112-113 mRNA stability assay. To know whether the poly(A) signals are important for the stability of M112-113 mRNA, we performed an mRNA stability assay according to previously reported protocol [25]. Briefly, we infected NIH3T3 cells with MCMVE5gfp_dpA3, MCMVE5gfp_dpA23, or MCMVE5gfp_dpA123 and their revertants at an MOI of 0.1. At the time of 20 h postinfection (hpi), 10 µg/mL actinomycin D was added to cells. The cells were collected for isolation of total RNA at time points of 1, 2, 4, and 8 h, following the actinomycin D addition. RT-qPCR was performed, as described above, for the M112-113 gene and GAPDH (Table 3). The levels of M112-113 mRNA were normalized with GAPDH, then the normalized M112-113 mRNA levels of MCMVE5gfp_dpA3, MCMVE5gfp_dpA23, or MCMVE5gfp_dpA123 were compared to the revertant of each mutated MCMV. A *t*-test was used to compare the differences between the groups, * *p* < 0.05, and ** *p* < 0.01.

Generally, a poly(A) signal is needed for stabilizing mRNA [25,26]. We asked whether the poly(A) of M112-113 stabilizes mRNA. To answer that question, we performed an mRNA-stabilizing experiment. MCMVE5gfp-dpA3, MCMVE5gfp-dpA23, or MCMVE5gfp-dpA123 or the respective revertant (MCMVE5gfp-dpA3RV, MCMVE5gfp-dpA23RV, or MCMVE5gfp-dpA123RV) was used to infect NIH3T3 cells at an MOI of 0.1 for 20 h. Then, 10 μg/mL actinomycin D was added to cells to stop the transcription. We isolated the total RNA at time points of 1, 2, 4, and 8 h following the treatment of actinomycin D. Lastly, the RT-qPCR experiments were performed using primers for the M112-113 gene and GAPDH (Table 3). The levels of M112-113 mRNA were normalized with GAPDH. Then, the normalized M112-113 mRNA levels of MCMVE5gfp_dpA3, MCMVE5gfp_dpA23, or MCMVE5gfp_dpA123 were compared to that of the respective revertant. The ratios are shown for each virus at different time points in Figure 7. As can be seen, the ratios of mRNA levels between groups of MCMVdPA3 and MCMVdpA23 to their respective revertant are around 1 and have no significant changes following the time of actinomycin D treatment, which implies that deleting one or 2 poly(A) signals has no effect on M112-113 mRNA stability. However, the ratio of the M112-113 mRNA level of MCMVdpA123 to that of its revertant declines following the time of treatment with actinomycin D, suggesting that the deletion of all the poly(A) signals made the M112-113 poly(A) unstable.

## 4. Discussion

Human cytomegalovirus (HCMV) belongs to the beta-subfamily of human herpesvirus, and it infects people of all ages. Cytomegalovirus (CMV) remains dormant within the bodies of healthy individuals after primary infection, and the latent CMV can reactivate to cause diseases in immunodeficient populations such as neonates, AIDS patients, and organ transplant recipients. CMV encodes more than 170 genes, and its gene expression consists of three tightly coordinated and regulated phases: the immediate-early phase, early phase, and late phase. The immediate-early gene products, along with cellular proteins, are required to activate early gene expression. One early gene product of interest is the early 1 (E1) protein, which is encoded by the UL112-113 gene region in HCMV or the M112-113 gene region in murine CMV (MCMV). The M112-113 gene consists of four isoforms that are produced by alternative splicing: pp87, pp38, pp36, and pp33. Interestingly, the M112-113 transcript consists of three polyadenylation (poly(A)) signals that are very close, which form a poly(A) cluster. However, the significance of the poly(A) cluster on M112-113 protein production and, ultimately, CMV replication has yet to be determined.

Our objective in this study was to generate mutations in the poly(A) cluster to be used to identify which poly(A) signal is important for M112-113 gene expression. Five different deletion mutations of the poly(A) signal cluster and the 3′UTR region of the M112-113 gene were generated, as shown in Figure 1. Deletion mutations were completed by PCR based on an M112-113 expressing plasmid, pBB2.9. The pBB2.9 plasmid contains the whole M112-113 gene, including promoter, exons, introns, and poly(A). According to the Western blot (Figure 2) and immunofluorescence assay (Figure 1 and Figure 2), it was discovered that one out of the three poly(A) signals is sufficient for M112-113 gene expression after transfection.

The second major finding is that the poly(A) signal cluster of the M112-113 gene is not required for MCMV replication. However, the deletion of all three poly(A) signals resulted in a significant decrease in viral protein production and viral growth (Figure 5 and Figure 6). When only one or two poly(A) was deleted, M112-113 gene expression was not affected in the context of both plasmid-based transfection and MCMV infection. Therefore, the poly(A) signal cluster is not required, and only one poly(A) signal of the M112-113 gene is sufficient for MCMV to replicate. We also found that the deletion of all the 3 PASs made the M112-113 mRNA relatively unstable (Figure 7). The reduction of viral replication and gene expression after the deletion of all the poly(A) signals might be caused by the lessened stability of the mRNA of M112-113.

Lastly, we found that the poly(A) signal cluster is not essential for IE3 to activate M112-113 gene expression. For example, as shown in Figure 2 and Figure 3, IE3 cotransfection with different M112-113 plasmids significantly increased the production of M112-113 gene products at both transcriptional and translational levels. However, the activation of the M112-113 gene by IE3 was attenuated significantly when all the three poly(A) signals were deleted. Since IE3 activation is through its interaction with the M112-113 gene promoter [21], it implies that one of the poly(A) signals might be needed to stabilize the mRNA to be exported to the cytoplasm for translation.

We recently demonstrated that IE3 interacts with cellular and viral proteins, eliminates repressive effects of cellular gene suppressors on viral gene expression, has a strong effect on early gene regulation, and is critical to the formation of viral prereplication compartments (pre-RC) via interactions with viral and cellular proteins [9,22]. In another attempt to determine how IE3 activates early gene expression, we identified a 10-bp cis-regulating motif, upstream of the M112-113 TATA box, as important for IE3 activation of M112-113 expression [21]. In addition, IE3 interacts with the TATA box binding protein (TBP), a core protein of TFIID (transcription initiation) complexes. We previously found that the interactions of IE3 with the 10-bp DNA element and with TBP stabilize the TFIID complex at the early gene promoter, such that M112-113 gene expression can be activated and/or enhanced. Here, we identified that a poly(A) signal of the M112-113 gene is sufficient to stabilize the IE3-activated M112-113 transcripts. Interestingly, none of the poly(A) signals are required for M112-113 gene expression and MCMV replication.

In summary, we have discovered that the 3-poly(A) cluster in the M112-113 gene is not essential for M112-113 gene expression and can be deleted for MCMV replication. Deletion of all three poly(A)s attenuates MCMV replication, but any one of the triple poly(A)s is sufficient for MCMV to replicate as wild-type MCMV. It is still mysterious why MCMV carries a 3-poly(A) cluster at the end of the M112-113 gene. Whether or not it affects its pathogenesis in vivo needs to be tested experimentally in the future.

## Figures and Tables

**Table 1:** Primers used for mutating poly(A) cluster at the end of the M112-113 gene.

E1ClaI	TTATCGATCGCCTCGTCTCGCT
PstIe1rev	TGGGGGCTGCAGGCTCAGAAGAAGGAATAC
E1PstIdlPA123_Rv	tgggggctgcagACATGGATGTTATGTTGTTAGC
E1PstIdlPA23_Rv	tgggggctgcagGATAAAATATTAAAAAATTAA
E1PstIdlPA12-Rv	tgggggctgcagCGTGTTTATTGTAACAAATCTC
E1PstIdlPA3_Rv	tgggggctgcagGTAACAAATGTGTGGTTTTT
E1PstIdlPA0_Rv	tgggggctgcagTGTTTATTGTAACAAATGTG

**Table 2:** Primers for bacterial artificial chromosome (BAC) mutagenesis to generate the mutations of M112-113.

E1polyA_galK_F	TGTGTGTCTCTGTCTACCGGGCCGATGGAGATATTATCCCTGGTCCCCCTCT CCTGTTGACAATTAATCATCGG CA
E1polyA_galK_R	TAACCACCGATGAGAATAATATTATTATCTCCCCCACAACCTCCTCCAACTC TCAGCACTGTCCTGCTCCTT
E1polyA_F	TGTGTGTCTCTGTCTACCG
E1polyAdpA123_R	TAACCACCGATGAGAATAATATTATTATCTCCCCCACAACCTCCTCCAACTC ggg ctg cag aca tgg atg
E1polyAdpA23_R	TAACCACCGATGAGAATAATATTATTATCTCCCCCACAACCTCCTCCAACTC ggg ctg cag gat aaa ata
E1polyAdpA3_R	TAACCACCGATGAGAATAATATTATTATCTCCCCCACAACCTCCTCCAACTC ggg ctg cag gta aca aat

**Table 3:** Primers for real-time RT-PCR of M112-113.

M112-113-Forward	ACT GCA CGA ACG GCG AGG
M112-113-Reverse	CAG GAT GAC TTG CAG CAA
GAPDH-Forward	GAAGGTGAAGGTCGGAGTC
GAPDH-Reverse	GAAGATGGTGATGGGATTTC

## References

[1] Reddehase M.J., Simon C.O., Seckert C.K., Lemmermann N., Grzimek N.K. (2008). Murine model of cytomegalovirus latency and reactivation. Curr. Top. Microbiol. Immunol..

[2] Mocarski E.S., Shenk T., Griffins P.D., Pass R.F. (2013). Cytomegaloviruses.

[3] Buhler B., Keil G.M., Weiland F., Koszinowski U.H. (1990). Characterization of the murine cytomegalovirus early transcription unit e1 that is induced by immediate-early proteins. J. Virol..

[4] Sarisky R.T., Hayward G.S. (1996). Evidence that the UL84 gene product of human cytomegalovirus is essential for promoting oriLyt-dependent DNA replication and formation of replication compartments in cotransfection assays. J. Virol..

[5] Pari G.S., Anders D.G. (1993). Eleven loci encoding trans-acting factors are required for transient complementation of human cytomegalovirus oriLyt-dependent DNA replication. J. Virol..

[6] Arai Y., Ishiwata M., Baba S., Kawasaki H., Kosugi I., Li R.Y., Tsuchida T., Miura K., Tsutsui Y. (2003). Neuron-specific activation of murine cytomegalovirus early gene e1 promoter in transgenic mice. Am. J. Pathol..

[7] Wells R., Stensland L., Vieira J. (2009). The human cytomegalovirus UL112–113 locus can activate the full Kaposi’s sarcoma-associated herpesvirus lytic replication cycle. J. Virol..

[8] Schumacher U., Handke W., Jurak I., Brune W. (2010). Mutations in the M112/M113 coding region facilitate murine cytomegalovirus replication in human cells. J. Virol..

[9] Tang Q., Li L., Maul G.G. (2005). Mouse cytomegalovirus early M112/113 proteins control the repressive effect of IE3 on the major immediate-early promoter. J. Virol..

[10] Ciocco-Schmitt G.M., Karabekian Z., Godfrey E.W., Stenberg R.M., Campbell A.E., Kerry J.A. (2002). Identification and characterization of novel murine cytomegalovirus M112–113 (e1) gene products. Virology.

[11] Cizkova D., Baird S.J.E., Tesikova J., Voigt S., Ludovit D., Pialek J., de Bellocq J.G. (2018). Host subspecific viral strains in European house mice: Murine cytomegalovirus in the Eastern (Mus musculus musculus) and Western house mouse (Mus musculus domesticus). Virology.

[12] Schwartz R., Helmich B., Spector D.H. (1996). CREB and CREB-binding proteins play an important role in the IE2 86-kilodalton protein-mediated transactivation of the human cytomegalovirus 2.2-kilobase RNA promoter. J. Virol..

[13] Lang D., Gebert S., Arlt H., Stamminger T. (1995). Functional interaction between the human cytomegalovirus 86-kilodalton IE2 protein and the cellular transcription factor CREB. J. Virol..

[14] Rodems S.M., Clark C.L., Spector D.H. (1998). Separate DNA elements containing ATF/CREB and IE86 binding sites differentially regulate the human cytomegalovirus UL112–113 promoter at early and late times in the infection. J. Virol..

[15] Arlt H., Lang D., Gebert S., Stamminger T. (1994). Identification of binding sites for the 86-kilodalton IE2 protein of human cytomegalovirus within an IE2-responsive viral early promoter. J. Virol..

[16] Spector D.H. (1996). Activation and regulation of human cytomegalovirus early genes. Intervirology.

[17] Wen F., Armstrong N., Hou W., Cruz-Cosme R., Obwolo L.A., Ishizuka K., Ullah H., Luo M.H., Sawa A., Tang Q. (2019). Zika virus increases mind bomb 1 levels, causing degradation of pericentriolar material 1 (PCM1) and dispersion of PCM1-containing granules from the centrosome. J. Biol. Chem..

[18] Hengel H., Lucin P., Jonjic S., Ruppert T., Koszinowski U.H. (1994). Restoration of cytomegalovirus antigen presentation by gamma interferon combats viral escape. J. Virol..

[19] Wu C.A., Carlson M.E., Henry S.C., Shanley J.D. (1999). The murine cytomegalovirus M25 open reading frame encodes a component of the tegument. Virology.

[20] Loh L.C., Keeler V.D., Shanley J.D. (1999). Sequence requirements for the nuclear localization of the murine cytomegalovirus M44 gene product pp50. Virology.

[21] Perez K.J., Martinez F.P., Cosme-Cruz R., Perez-Crespo N.M., Tang Q. (2013). A short cis-acting motif in the M112–113 promoter region is essential for IE3 to activate M112–113 gene expression and is important for murine cytomegalovirus replication. J. Virol..

[22] Martinez F.P., Cosme R.S., Tang Q. (2010). Murine cytomegalovirus major immediate-early protein 3 interacts with cellular and viral proteins in viral DNA replication compartments and is important for early gene activation. J. Gen. Virol..

[23] Wagner M., Jonjic S., Koszinowski U.H., Messerle M. (1999). Systematic excision of vector sequences from the BAC-cloned herpesvirus genome during virus reconstitution. J. Virol..

[24] Warden C., Tang Q., Zhu H. (2011). Herpesvirus BACs: Past, present, and future. J. Biomed. Biotechnol..

[25] Ratnadiwakara M., Änkö M. (2018). mRNA Stability Assay Using Transcription Inhibition by Actinomycin D in Mouse Pluripotent Stem Cells. Bio-Protocol.

[26] Colgan D.F., Manley J.L. (1997). Mechanism and regulation of mRNA polyadenylation. Genes Dev..

[27] Beaudoing E., Freier S., Wyatt J.R., Claverie J.M., Gautheret D. (2000). Patterns of variant polyadenylation signal usage in human genes. Genome Res..

